# Plankton metacommunities in floodplain wetlands under contrasting hydrological conditions

**DOI:** 10.1111/fwb.13076

**Published:** 2018-02-07

**Authors:** Griselda Chaparro, Zsófia Horváth, Inés O'Farrell, Robert Ptacnik, Thomas Hein

**Affiliations:** ^1^ WasserCluster Lunz Lunz am See Austria; ^2^ Instituto de Ecología, Genética y Evolución de Buenos Aires Universidad de Buenos Aires – Consejo Nacional de Investigaciones Científicas y Técnicas Buenos Aires Argentina; ^3^ Institute of Hydrobiology and Aquatic Ecosystem Management University of Natural Resources and Life Sciences Vienna Austria

**Keywords:** beta‐diversity, environmental heterogeneity, phytoplankton, spatial scale, zooplankton

## Abstract

1. Species diversity is affected by processes operating at multiple spatial scales, although the most relevant scales that contribute to compositional variation and the temporal shifts of the involved mechanisms remain poorly explored. We studied spatial patterns of phytoplankton, rotifers and microcrustacean diversity across scales in a river floodplain system of the Danube in Austria under contrasting hydrological conditions (post‐flood versus low water level).
2. The species turnover between water sections (β2) and between wetlands (β3) was the major components of regional diversity for all studied groups, with species turnover between habitats (β1) as a minor contributor. β1 diversity and β2 diversity were lower than expected by chance in most cases, suggesting that communities are more homogeneous than expected at these scales. β3 diversity was higher than expected by chance in many cases, indicating more distinct communities at the wetland level. Patterns were highly similar under different hydrological conditions, indicating no major immediate effect of flood events.
3. Local environmental and spatial factors were similarly important in structuring phytoplankton, rotifer and microcrustacean communities in both hydrological conditions. Relevant environmental factors were spatially structured in post‐flood conditions especially between sections, suggesting flood‐driven homogenisation within the wetlands. Under low water level, spatial structuring of environment decreased and pure environmental factors gained relevance for phytoplankton and rotifers.
4. Our results suggest that although β2 diversity between water sections is a major component of regional diversity, long‐term spatial processes responding to connectivity across the wetland structure phytoplankton, rotifer and microcrustacean communities. Aquatic sections within the limited spatial extent of the remaining floodplain areas appear more homogeneous than expected probably due to flood recurrence over the years.
5. These results highlight that adequate planning of restoration and conservation strategies of floodplain wetlands should consider environmental heterogeneity together with long‐term spatial processes.

## 1 INTRODUCTION

Understanding the spatial distribution of species diversity is a major topic in ecology, and its relevance is enhanced under the current scenario of habitat alteration, fragmentation and progressive diversity loss (Gaston, 2000; McGill, Dornelas, Gotelli, & Magurran, 2015; Pinel‐Alloul et al., 2013). The total species diversity in a certain region (γ‐diversity) can be split into the number of species from a local community (α‐diversity) and the species turnover (β‐diversity), which represents the variation in species composition among localities within the region (Whittaker, 1972). Measures of species diversity, including β‐diversity, are dependent on the spatial scale considered. One reason for this is that environmental factors that affect species distribution have a different range of variation in space: some variables show large variation at small spatial extents and can cause high species turnover in small areas, while others change at larger scales and then associated turnover can only be detected at large spatial extent (Borcard, Legendre, Avois‐Jacquet, & Tuomisto, 2004; Declerck, Coronel, Legendre, & Brendonck, 2011; Levin, 1992). Dispersal of individuals strongly influences species distribution and their relationship with the local environment and varies with the spatial scale (Heino, Melo, & Bini, 2015; Leibold et al., 2004; Ng, Carr, & Cottenie, 2009). At intermediate scales, where moderate dispersal rates are likely to occur, most individuals are able to reach suitable sites and local environmental factors would be the most important in shaping communities composition, supporting the *species sorting* metacommunity model (Heino, Melo, Siqueira, et al., 2015). In this scenario, a positive relation between β‐diversity and environmental heterogeneity can be detected, because an increase in the latter incorporates an increase in the variety of environmental conditions to which different species are adapted, hence producing greater variation in species composition among localities within a region (Heino, Melo, & Bini, 2015; Leibold et al., 2004). At small spatial extent, high dispersal rates can lead to *mass effects*, where the continuous dispersal of organisms from favourable “source” sites determines the presence of species in unfavourable “sink” sites, thus homogenising the communities. Conversely, at broader scales, dispersal limitation is more likely to occur, preventing a large proportion of individuals of reaching favourable sites and provoking a high β diversity (Heino, Melo, Siqueira, et al., 2015). Both mass effects and dispersal limitation weaken the match between local communities and environmental conditions (Heino, Melo, & Bini, 2015) and produce significant spatial structuring of the communities (Ng et al., 2009), thus making it hard to differentiate their effects in field studies. One useful strategy to disentangle them is to focus on nested spatial scales, where significant effects at small spatial extent would represent mass effects and significant effects at large spatial extent would suggest dispersal limitation (Dray et al., 2012; Heino, Soininen, Alahuhta, Lappalainen, & Virtanen, 2016).

Riverine floodplains host exceptionally high biodiversity attributed to high spatial and temporal heterogeneity (Junk, Bayley, & Sparks,^1989^; Tockner & Stanford, 2002). Here, the distinct habitats are hierarchically arranged in space, which allows studying nested spatial patterns of diversity (Tockner, Lorang, & Stanford, 2010; Ward, Tockner, Arscott, & Claret, 2002). Understanding the various mechanisms that shape species distribution is especially challenging in these dynamic systems, because local environmental conditions and habitat connectivity (which affects dispersal) are associated with the discharge regime of the river and vary continuously (Datry, Bonada, & Heino, 2016; Fernandes, Henriques‐Silva, Jerry, Jansen, & Peres‐Neto, 2014). In high water periods, the connectivity between environments increases, enhancing the exchange of sediment, minerals, substrates and organisms between different habitats (Thomaz, Bini, & Bozelli, 2007). In periods of low water, habitats are isolated from each other and from the main river channel and β‐diversity increases over time (Bozelli, Thomaz, Padial, Lopes, & Bini, 2015; Lansac‐Toha, Meira, Segovia, Lansac‐Toha, & Velho, 2016).

We studied spatial patterns of phytoplankton, rotifer and microcrustacean zooplankton (copepods, cladocerans) diversity in contrasting hydrological conditions (post‐flood, low water level) in a river floodplain system of the Danube River in Austria. The three taxonomic groups differ in body size, life cycle, sensitivity to environmental conditions and mobility and may exhibit different spatial patterns (De Bie et al., 2012; Padial et al., 2014). Rotifers are small and have short development times and fast population recovery from flushing effects, whereas larger microcrustaceans with longer growth rates are more sensitive to floods (Baranyi, Hein, Holarek, Keckeis, & Schiemer, 2002; Chaparro, Fontanarrosa, Schiaffino, de Tezanos Pinto, & O'Farrell, 2014; Costa Bonecker, Da Costa, Machado Velho, & Lansac‐Toha, 2005).

We used a multiscale approach: (1) *habitats* of different vegetation types, which represent a main source of environmental heterogeneity within water sections and a main driver for community composition (Thomaz & Ribeiro Da Cunha, 2010); (2) *water sections* within floodplain wetlands, along a gradient of lateral connectivity with the main channel, which determines main local environmental conditions affecting studied communities (e.g., turbidity, flow, nutrients, macrophyte cover) and dispersal potential (Thomaz et al., 2007); (3) three *wetlands* (distinct segments of the floodplain) located within the Donau‐Auen National Park river stretch, in a free‐flowing section of the Danube River in Austria that constituted the regional scale in this study.

The aims of this study were as follows: (1) to compare the relative contribution of species richness (α) and species turnover (β) to regional diversity (γ) in phytoplankton, rotifer and microcrustacean from riverine floodplains under contrasting hydrological conditions; (2) to explore the relationship between β diversity and environmental heterogeneity; and (3) to assess changes in the relative influence of local environmental and spatial factors (dispersal) on species composition under contrasting hydrological conditions.

More specifically, we tested the following hypotheses: (1) β‐diversity at all studied scales (*between habitats*,* between water sections* and *between wetlands*) are relevant components of phyto‐ and zooplankton γ diversities in riverine floodplains, especially under low water level conditions; (2) the relationship between β‐diversity and environmental heterogeneity is weak after a flood pulse and increases under low water level conditions; (3) floods homogenise environmental conditions and the plankton communities within the floodplain wetlands, weakening environmental control; while in low water conditions, local environmental factors are the main drivers of community composition.

## 2 METHODS

### 2.1 Study area

**Figure 1:**
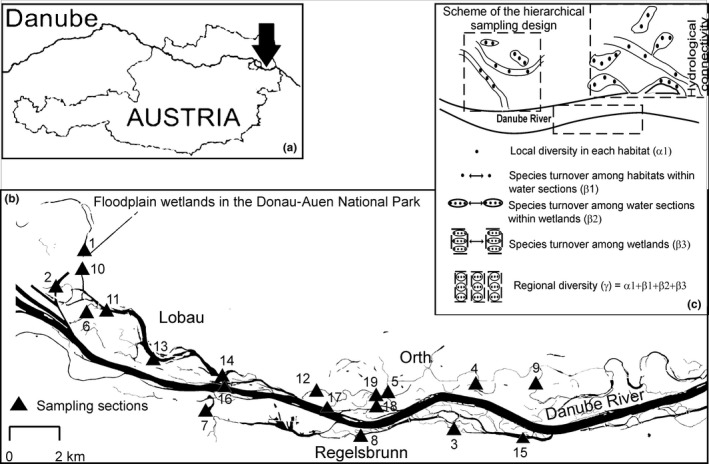
(a) Map with the geographic location of the Donau‐Auen National Park in Austria, indicated by the black arrow; (b) location of the floodplain wetlands included (Lobau, Orth and Regelsbrunn) and the selected sampling sections (triangles); (c) scheme of the hierarchical sampling design and levels of additive partition of diversity. Sampled sections are numbered from 1 to 19. Due to accessibility reasons, Section 1 was only included in 2014 and Section 2 only in 2015; both sections belong to the same low connectivity level

The Danube River is 2,900 km long and drains an area of 817,000 km^2^. At Vienna (Austria), its mean annual discharge is *c*. 1,950 m^3^/s and annual flood discharge above 5,800 m^3^/s. Historically braided, the floodplain has been constrained by major regulations that began in 1875. The floodplain area east of Vienna (93 km^2^) was given the status of a national park in 1996 (Schiemer, Baumgartner, & Tockner, 1999) Donau‐Auen National Park (Figure 1a). Following side‐arm restoration, the national park contains sections of differing hydrological connectivity, ranging from predominantly lentic waterbodies to areas of intermediate and high connectivity with the river main channel. It is among the last remnants of river‐floodplain systems in Central Europe (Schiemer et al., 1999; Tockner, Schiemer, & Ward, 1998). Three floodplain wetlands within the national park were selected in this study: Lobau, located within the city limits of Vienna; Regelsbrunn, situated downstream south‐east of the Lobau; and Orth, to the east just downstream of the Lobau (Figure 1b). Detailed characterisation of these wetlands (environmental conditions and hydrological connectivity patterns) can be found in previous studies (Baart, Gschöpf, Blaschke, Preiner, & Hein, 2010; Hein, Baranyi, Reckendorfer, & Schiemer, 2004).

### 2.2 Hierarchical sampling

**Figure 2:**
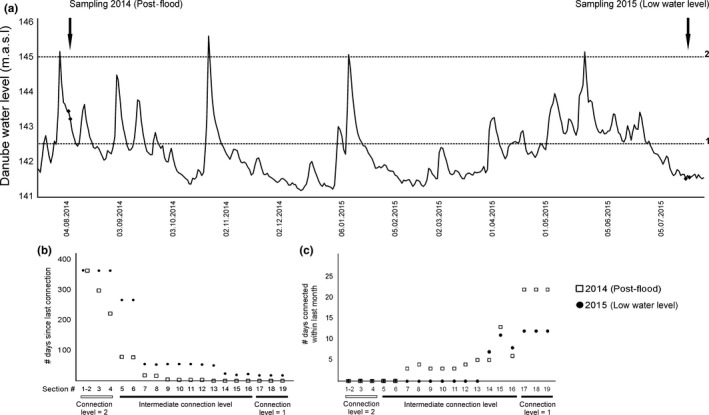
(a) Water level fluctuation of the Danube River at the Wildungsmauer gauge station (River—km 1882) within the Donau‐Auen National Park stretch, between summer 2014 and summer 2015 (m.a.s.l. = metres above sea level); black arrows indicate the two sampling periods and black diamonds indicate the sampling dates; 1—indicates the water level at which the floodplain wetlands get connected with the river (water sections located close to the Danube River get connected); 2—indicates the water level at which even the least connected sections in the floodplains are connected with the river. 1 and 2 were calculated following methods described by Welti et al. (2012). (b) Time elapsed (# days) since last connection for each section. (c) Number of days connected within the previous month to the sampling date for each section. For reference to section numbers, see Figure 1

We sampled phyto‐ and zooplankton communities in the national park at three nested spatial scales (Figure 1c): (1) *between habitats* (open waters, submerged macrophytes, floating‐leaved macrophytes and helophytes) in each water section whenever present; (2) *between water sections* along a gradient of hydrological connectivity with the main channel (ranging from 0 to more than 250 days/year) in each wetland; and (3) *between wetlands* (Lobau, Regelsbrunn and Orth). Vegetated habitats with a plant cover near 100% were selected to avoid possible differences associated with variations in vegetation cover. Water sections were selected to cover the full gradient of hydrological connectivity, which was estimated with a connectivity parameter defined as the average annual duration (days per year) of the surface connection of floodplain water sections with the Danube River (Reckendorfer, Baranyi, Funk, & Schiemer, 2006). To include the temporal changes associated with the river discharge regime, the sampling was performed twice at each site: once in summer 2014 within 3–10 days after a flood pulse that inundated 13 of the 18 water sections included in the sampling (flooded conditions), and once in summer 2015 during a low flow period when 12 of 18 water sections were disconnected from the river for *c*. 55 consecutive days and the remaining six sections with higher connectivity level were disconnected for *c*. 20 consecutive days (low water level condition) (Figure 2a,b; water level data provided by Water administration Lower Austria, Austria). Besides, water level dynamics determined an overall higher connectivity of the wetlands to the Danube River within the previous month to the sampling date in summer 2014 (Figure 2c). In total, 35 sampling sites were included in 2014 and 26 in 2015.

### 2.3 Phyto‐ and zooplankton

For phytoplankton, 300 ml of unfiltered water was taken from the subsurface at each sampling site, preserved with 1% Lugol's iodine solution and identified under microscope to the lowest possible taxonomic level using specialised literature (Supporting information) and counted according to Utermöhl (1958). Samples for zooplankton were taken at each sampling site with a transparent acrylic bottle (12 cm diameter, 100 cm long) adequate for both vegetated and open water areas (Paggi, Mendoza, Debonis, & José de Paggi, 2001). In each habitat, twenty litres of water were obtained by submerging the bottle several times and collecting 2–4 L of integrated water column from surface to bottom or to a maximum of 100 cm deep. The total volume (20 L) was filtered through a 40‐μm mesh sieve and preserved with 4% formaldehyde. Rotifers, adult copepods and cladocerans were identified to the lowest possible taxonomic level (mostly species, genus in a few cases) using specialised literature (Einsle, 1993; Flössner, 2000; Koste, 1978) under microscope. Rotifers were counted using 1‐ml Sedgwick–Rafter chambers under microscope and microcrustaceans using 5‐ml Bogorov chambers under stereomicroscope. In all cases, we refer to the identified taxa as species.

### 2.4 Environmental variables

Water temperature, pH, conductivity and dissolved oxygen (the latter only in the first sampling) were measured in situ in each sampling site using HQ40d Hach^®^ portable meter, water depth with a wooden meter and flow velocity with a Flo‐Mate 2000 (Marsh‐Mc Birney). Water samples were collected and immediately filtered using pre‐combusted GF/F (Whatman) fibreglass filters for dissolved nutrients, total dissolved organic carbon (DOC) and suspended solids (SS) analyses. Soluble reactive phosphorus was determined by the ascorbic acid reduction method, ammonium by the automated indophenol blue method and nitrate by the automated hydrazine reduction method (Eberlein & Kattner, 1987; Ivancic & Deggobis, 1984; Kempers & Luft, 1988) using a continuous flow analyser (CFA; Systea Analytical Technology) (ISO 13395:^1996^; [Link]ISO 15681‐2) An aliquot of 30 ml filtered sample was acidified 3% v/v using 2 m HCl, and DOC was analysed using a Sievers 900 Portable TOC Analyzer (GE Analytical Instruments). To determine SS, 100–1,000 ml of water were filtered through pre‐combusted GF/F filters and determined gravimetrically after drying at 103–105°C until constant weight (American Public Health Association 2005). Samples for chlorophyll‐*a* were filtered through Whatman GF/C filters and stored at −20°C for 24 hr, homogenised with a Polytron mixer (PT 1600E) and extracted with 5 ml cold 90% acetone overnight. After centrifugation (1370 G, 20 min), chlorophyll‐*a* content was determined spectrophotometrically (Jeffrey & Humphrey, 1975; Lorenzen, 1967).

### 2.5 Spatial factors

The spatial configuration of the sampling sites was represented by two components: the section component, describing the spatial relationships among the water sections within each wetland, and the wetland component representing wetland identity using dummy variables. The section component consisted of Moran's eigenvector maps, which produce orthogonal spatial variables derived from geographic coordinates of the sampling sites (Dray, Legendre, & Peres Neto, 2006). The eigenvector map variables were arranged in blocks, each block corresponding to one wetland, using the function *create.MEM.model* provided by Declerck et al. (2011). A forward selection procedure (permutation test with 999 simulations) was applied to select for significant section and wetland variables (*p* < .05) for each community data set (phytoplankton, rotifers and microcrustaceans) and hydrological condition using the R package *packfor* (Dray, 2013). Selected variables were included in our analyses as spatial predictors.

### 2.6 Data analyses

The environmental variables water depth, SS, DOC, soluble reactive phosphorus, nitrate and ammonium in both years and flow velocity in 2014 were cubic root‐transformed to normalise their distribution. All environmental variables were standardised with the range method using the function *decostand* in the R package vegan (Oksanen et al., 2013).

Species accumulation curves for each taxonomic group were drawn for each sampling time to check the representativeness of the regional diversity in the national park, using the function *specaccum* in the R package *vegan* (Oksanen et al., 2013). Diversity was partitioned into local species richness (number of species) at each sampling site (α), species turnover *between habitats* within sections (β1), species turnover *between sections* within wetlands (β2) and species turnover *between wetlands* (β3). The regional (national park) diversity was expressed as follows: γ = α + β1 + β2 + β3. Observed diversity values were compared with expected random values obtained from null models (r2dtable method with 999 simulations) (Crist, Veech, Gering, & Summerville, 2003) using the function *adipart* in the R package vegan (Oksanen et al., 2013).

Dispersion diagrams were plotted to assess the relationship between β‐diversity and environmental heterogeneity. Bray–Curtis index was used to estimate β‐diversity and Euclidean distance based on standardised physicochemical parameters measured to estimate environmental heterogeneity at each level of the hierarchical design.

To disentangle the roles of local environmental and spatial effects in determining community composition, variation partitioning based on distance‐based redundancy analyses (db‐RDA; Legendre & Anderson, 1999) was performed with the selected environmental variables, and section and wetland spatial factors for each group and hydrological condition using the R package *ape* (Paradis et al., 2004). Environmental factors for each group and hydrological condition were selected by a forward selection procedure as detailed for the selection of spatial factors; Chl*a* was excluded as explanatory variable for phytoplankton. The relevance of each factor was determined by the adjusted *R* ^2^ values, which are independent of sample size and allow for comparisons between results (Peres‐Neto, Legendre, Dray, & Borcard, 2006). Variation partitioning analyses were performed on both presence–absence and abundance data.

## 3 RESULTS

The selected sampling sites covered a broad range of environmental conditions in both post‐flood and low water level conditions (Table 1). Flow velocity and SS concentration were remarkably higher in the post‐flood sampling. Most of the variables showed similar coefficient of variation (CV) between years, except for nitrate and phosphate concentrations and water depth, with higher values in low water level conditions.

**Table 1:** Summary of the environmental parameters measured in the three wetlands in post‐flood and low water level conditions. DOC, dissolved organic carbon; *SD*, standard deviation; CV, coefficient of variation; NA, not available

	Post‐flood (2014)				Low water (2015)			
	Range	Mean	*SD*	CV	Range	Mean	*SD*	CV
Water depth (m)	0.31–3	1.01	0.65	0.64	0.17–3	0.80	0.74	0.93
Water temperature (°C)	14.8–24.9	21.59	2.80	0.13	18–25	21.88	2.36	0.11
pH	7.46–8.87	8.02	0.37	0.05	7.08–8.64	7.96	0.38	0.05
Conductivity (mS/cm)	274–702	418.66	110.61	0.26	161–757	463.00	141.99	0.31
Dissolved oxygen (mg/L)	4.94–16.35	8.94	2.75	0.31	NA	NA	NA	NA
Flow velocity (m/seg)	0–1.88	0.10	0.37	3.50	0	0	0	0
Chlorophyll‐*a* (μg/L)	1.30–35.34	12.14	9.73	0.80	0.64–30.42	10.08	8.53	0.85
Suspended solids (mg/L)	0–148.12	23.79	39.15	1.65	0.62–67.55	11.25	14.43	1.28
DOC (mg/L)	1.17–9.34	3.73	1.98	0.53	2.11–13.03	4.93	2.69	0.55
N‐NH_4_ (μg/L)	4–175.4	27.37	28.64	1.05	6.1–144.3	33.00	35.06	1.06
N‐NO_3_ (μg/L)	0.1–2,187.2	384.38	503.22	1.31	12.5–3,362	292.44	893.20	3.05
P‐PO_4_ (μg/L)	0–25.4	7.22	9.51	1.32	0.1–130.2	10.47	28.33	2.71

The regional diversity was composed of 200 and 230 phytoplankton, 92 and 89 rotifers, and 35 and 32 microcrustacean species in post‐flood (2014) and in low water level (2015) conditions, respectively (Supporting information). Species accumulation curves started to stabilise from site 30 in post‐flood and from site 20 in low water level conditions; the curves were closer to saturation for microcrustaceans than for rotifers and phytoplankton (Figure S1).

**Figure 3:**
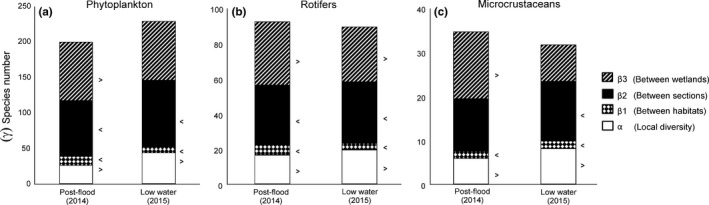
Results of the additive partition of regional diversity (richness) in the Donau‐Auen National Park in Austria for (a) phytoplankton; (b) rotifers and (c) microcrustaceans in post‐flood and low water level conditions. Symbols < and > denote significant differences compared to random values after 999 permutations (*p* < .05)

The additive partitioning of diversity showed similar results for the studied groups and no major differences between post‐flood and low water level conditions: β2 (*between sections*) and β3 (*between wetlands*) were the major contributors, followed by α, while β1 (*between habitats*) was always the smallest contributor. Null model analyses showed that β1 and β2 were lower than expected by chance in most cases (*p* < .05; Figure 3), suggesting rather homogeneous communities at these scales. Conversely, β3 was higher than expected by chance for all groups in post‐flood conditions and only for rotifers in low water level conditions (*p* < .05), indicating more distinction between the communities from the three wetlands. α was higher than expected by chance in all cases (*p* < .05).

**Figure 4:**
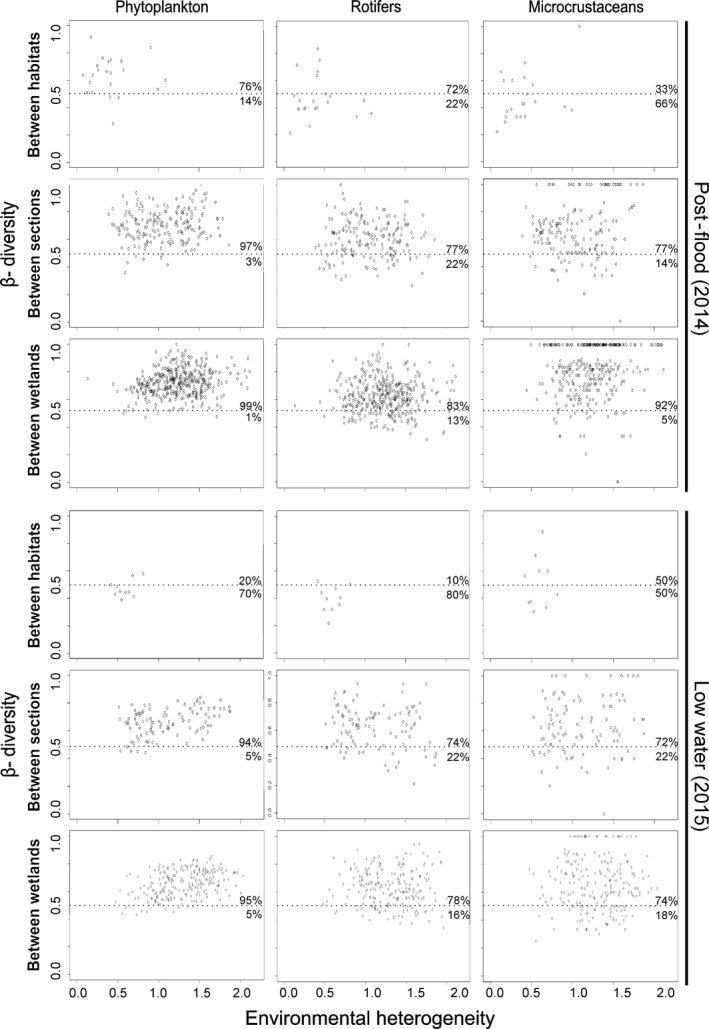
Dispersion diagrams of β‐diversity (Bray–Curtis index) and environmental heterogeneity (Euclidean distance) for each taxonomic group, spatial scale and hydrological condition (based on site by site pairwise comparisons). The proportion of values above and below a beta‐diversity value of .5 is presented in each subfigure

In agreement with results from additive partitioning of diversity, in the dispersion diagrams the proportion of values above a similarity index of 0.5 indicated higher β‐diversity values at the *between‐sections* and *between‐wetlands* scales for all studied groups (Figure 4). Environmental heterogeneity showed a similar pattern, with higher similar values *between sections* and *between wetlands* than *between habitats*. No associations were found between environmental heterogeneity and community dissimilarity, as clearly depicted by the high dispersion in all plots (Figure 4).

The concentrations of dissolved nutrients and DOC were the local environmental factor more frequently selected by the forward selection procedure for the studied communities in post‐flood and low water level conditions (presence‐absence data); Chl*a* was always selected for rotifers and microcrustaceans. From 2 to 3 spatial variables at the section scale were selected for each community in post‐flood, while 1 or 2 were selected in low water level; 1 or 2 spatial variables at the wetland scale were selected for all communities in post‐flood and only for rotifers in low water level conditions.

**Figure 5:**
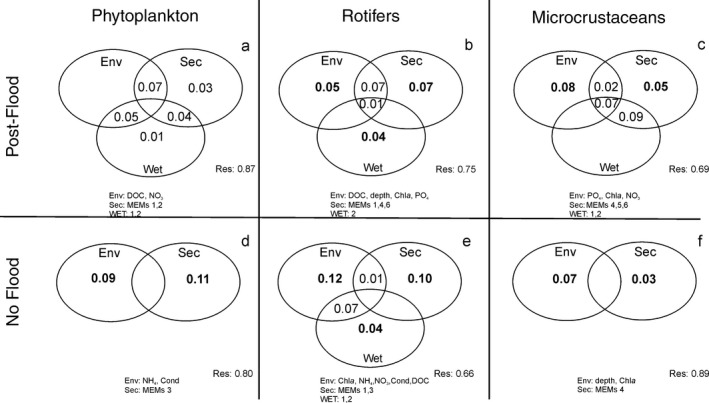
Results of the variation partitioning (distance‐based redundancy analyses, db‐RDA) of community composition (presence–absence data) into environmental (Env), spatial factors at the section (Sec) and wetland scales (Wet) in post‐flood (a–c) and low water level (d–f) conditions. The contribution of each factor is represented by *R* ^2^ adjusted values and bold numbers indicate significant effects (*p* < .05). Negative values are not shown

The results of variation partitioning based on presence–absence data showed that, during post‐flood conditions, environmental variables selected for each community were spatially structured especially at the section scale, as depicted by high amount of the shared environment‐section fraction (Figure 5a–c). No significant pure effects were detected for phytoplankton. Pure environmental and pure section effects were significant and explained similar amounts of variation in rotifers (5% and 7%, respectively) and microcrustaceans (8% and 5%, respectively); pure wetland effects were significant only for rotifers, accounting for 4% of the variation. In low water level, environmental conditions were not spatially structured, except for rotifers at the wetland scale. Pure environmental and pure section effects were significant and respectively explained 9% and 11% of the variation in phytoplankton, 12% and 10% in rotifers and 7% and 3% in microcrustaceans.

The variation partitioning based on abundance data showed, in post‐flood conditions, stronger environmental effects for all groups when compared to presence–absence data, though spatial effects (section + wetland) were very important in all cases (Supporting information). Similar results were obtained for phytoplankton in low water level conditions, with stronger environmental effects compared to the presence–absence data and similar contribution by environmental and spatial factors. It was not possible to perform the variation partitioning based on rotifer and microcrustacean abundances under low water level conditions because of lack of significant variables for at least two of the three groups of factors (only section variables were selected for rotifers and only environmental variables for microcrustaceans).

## 4 DISCUSSION

Species diversity is affected by processes operating at multiple spatial scales (Leibold et al., 2004), although the most relevant scales that contribute to compositional variation and the temporal shifts of the involved mechanisms are still to be identified (Datry et al., 2016; Dray et al., 2012; Heino et al., 2016).

We identified β‐diversities at intermediate (*between sections*) and broad (*between wetlands*) scales as the most relevant components for phytoplankton, rotifer and microcrustacean regional diversities in riverine floodplains of the Danube during contrasting hydrological conditions. The *between‐sections* scale comprised the gradient of hydrological connectivity of the floodplain habitats with the river, which is one of the most remarkable features of these systems, because it largely influences local environmental conditions that affect communities (Heiler, Hein, Schiemer, & Bornette, 1995; Tockner, Malard, & Ward, 2000). Depending on the hydrological connectivity with the river, floodplain habitats range from lotic, turbid, nutrient rich and frequently disturbed to lentic, clear vegetated conditions. Previous studies found that along such gradient, phytoplankton changed from taxa adapted to turbulent waters to those adapted to more stable conditions (Devercelli, 2014; Gallardo, Gascón, González‐Sanchís, Cabezas, & Comín, 2009; Schagerl, Drozdowski, Angeler, Hein, & Preiner, 2009); likewise, a shift from small fast growing to large (Baranyi et al., 2002) and from pelagic filter feeding to scraping zooplankton taxa associated with macrophytes was reported (Van den Brink, Van Katwijk, & Van der Velde, 1994). Our results regarding the great relevance of β2 (*between sections*) as component of regional diversity agree with these expectations, as this spatial scale comprises the main environmental gradient in dynamic floodplains. However, lower β2 values compared to random values indicate homogeneous communities at this scale, probably resulting from high connectivity promoted by recurrent flooding events, as those occurred during summer 2014 and late autumn 2015. The three wetlands (Lobau, Orth and Regelsbrunn) are still rather disconnected from each other despite restoration measures (Schiemer et al., 1999; Tockner et al., 1998). Habitat fragmentation together with environmental heterogeneity at the broad *between‐wetlands* scale might have contributed to the high values of β3 despite relatively short distances among these three wetlands. The finest *between‐habitats* scale comprised very distinct habitats, including open water areas and vegetated patches with distinct macrophyte life forms that were expected to provide differential niches regarding environmental conditions, refuge area and food availability (Ferreiro, Giorgi, & Feijoo, 2013; Thomaz & Ribeiro Da Cunha, 2010; Warfe & Barmuta, 2004). A shift from primarily epiphytic and littoral species in macrophyte stands to pelagic phyto‐ and zooplankton taxa in open waters was reported in previous studies (Avigliano, Vinocur, Chaparro, Tell, & Allende, 2014; Chaparro, Kandus, & O'Farrell, 2015; Duggan, Green, Thompson, & Shiel, 2001; José de Paggi et al., 2012). At the same time, these habitats are well connected and comprise one waterbody (section). This counteracted expectation related to the strong community shaping role of macrophyte stands, as we found low environmental heterogeneity coupled with low β1‐diversity values at this scale. This indicated that both environment and plankton communities were similar between different habitats within sections. Although our sampling collection method is especially suitable for shallow vegetated waterbodies (Paggi et al., 2001), an additional effect of possible undersampling specialised species in each habitat cannot be fully excluded.

Few studies have previously examined spatial patterns of diversity across spatial scales in floodplains. Simões et al. (2013) studied zooplankton diversity in floodplains from the Upper Paraná River, Matsuda et al. (2015) analysed ostracod diversity, and Dittrich, Deo Dias, Costa Bonecker, Lansac‐Tôha, and Padial (2016) analysed different communities in the same system. In general, our findings agree with their results and highlight the great relevance of the *between‐sections* scale, where species replacement in space is detected even in scenarios of high connectivity, such as during flooding and after flood pulses. However, the stability of the general patterns we found under contrasting hydrological conditions implies that floods have no instantaneous effect on the communities and that spatial structuring acts rather at longer time scales for the plankton biodiversity in these wetland systems.

While the significance of local environmental conditions and niche differences of species have been a central topic in studies on communities from riverine floodplains, spatial mechanisms were only recently considered and proved to be important for different communities, including molluscs, fish and macroinvertebrates (Fernandes et al., 2014; Funk, Schiemer, & Reckendorfer, 2013; Padial et al., 2014; Tonkin, Stoll, Jähnig, & Haase, 2016). In our study, both local environmental and spatial processes were important in structuring phytoplankton, rotifer and microcrustacean communities, supporting the idea of a continuum between different mechanisms influencing community assembly (Logue, Mouquet, Hannes, & Hillebrand, 2011). The comparison between post‐flood and low water level conditions revealed little temporal shifts in the relative importance of local and spatial factors, which were linked to the discharge regime of the river (Datry et al., 2016; Fernandes et al., 2014). Relevant environmental factors were spatially structured in post‐flood conditions especially between sections, suggesting flood‐driven homogenisation within the wetlands. In low water level, spatial structuring decreased leading to enhanced relevance of pure environmental factors for phytoplankton and rotifers. Regarding microcrustaceans, the overall very low explained variability implies that other than measured variables influence their composition during low water level periods. Although a recent study has shown an increase in the importance of environmental and spatial predictors to explain the distribution of microorganisms in the low water period (Dias et al., 2016), previous studies in floodplains from the Danube found that, during periods of low water level and long residence time, biotic interactions gained relevance over abiotic factors (Baranyi et al., 2002). Predation by zooplanktivorous fish is a main driving factor for zooplankton communities, especially affecting microcrustaceans (Sinistro, 2010). Although there are no available data about predation pressure on zooplankton for these floodplains, their potential role cannot be discarded.

Variation partitioning based on abundance data showed similar general patterns; the overall higher explained variability and higher relevance of pure environmental factors than those obtained with analyses based on presence–absence data indicate a better explanation of the relationship between community composition and their habitats, which is a frequent pattern in metacommunities (Anderson et al., 2011; Heino, 2014).

Previous studies from the Upper Paraná floodplains suggested the main drivers of community composition remained through the hydrological temporal variability (Dittrich et al., 2016; Padial et al., 2014). In the Danube River, floods occurring almost every year reach most of the aquatic habitats within the floodplain wetlands at moderate frequencies (Reckendorfer et al., 2006). In fact, although our sampling design covered different connectivity conditions of the sampling sites, several floods took place in between our sampling period and attenuated the differences and accounted for the similar patterns registered in both periods. Floods might not only transport and homogenise active communities, but also the resting stages deposited in sediments (Simões et al., 2013; Vaníčková, Seda, Macháček, & Petrusek, 2011). Resting stages are extremely abundant and diverse in floodplain habitats (Shiel, Green, & Tan, 2001) and are an important source of organisms that contribute to phyto‐ and zooplankton dynamics (Jenkins & Boulton, 2003; Reynolds & Descy, 1996) and are considered to act as a system memory (Dittrich et al., 2016). This supports the implications of our findings regarding the dominance of long‐term effects of connectivity on the communities. The regular occurrence of homogenising flood events also explains null or weak relationships between β‐diversity and environmental heterogeneity (Heino & Grönroos, 2013; Heino, Melo, & Bini, 2015).

The relevance of habitat heterogeneity and niche differences of species have been a central paradigm in ecological studies on communities from riverine floodplains. The importance of spatial processes has become increasingly clear in recent decades with the understanding of their roles in promoting differential structuring of diversity depending on the scale (Da Silva & Medina Hernández, 2015). Our results suggest that although β2‐diversity between water sections within wetlands is a major component of the regional diversity, long‐term spatial processes tend to structure phytoplankton, rotifers and microcrustaceans communities at this scale. This is probably related to the regular occurrence of floods that connect most aquatic habitats within the limited spatial extent of the remaining floodplain areas compared to pristine systems and has important implications for the diversity in the long‐term perspective. These results highlight that adequate planning of restoration and conservation strategies of floodplain wetlands in highly altered riverine landscapes should consider environmental heterogeneity together with long‐term spatial effects.
