# Supplementary material for: Plankton metacommunities in floodplain wetlands under contrasting hydrological conditions
Source: Freshw Biol. 2018 Feb 7;63(4):380–91. doi: 10.1111/fwb.13076 (PMC5993336; doi:10.1111/fwb.13076)
Supplement: Supplementary file 3 [file FWB-63-380-s003.docx]

Table S1. List of identified phytoplankton, rotifer and microcrustacean species in the Donau Auen National Park in our study. X indicates the presence of the species in each sampling date (2014, Post-flood and 2015, Low water)

|  | **Post-flood** | **Low water** |
| --- | --- | --- |
| ***PHYTOPLANKTON*** |  |  |
| **Cyanobacteria** |  |  |
| *Aphanocapsa delicatissima* | X | X |
| *Aphanocapsa holsatica* |  | X |
| *Aphanocapsa incerta* | X | X |
| *Aphanocapsa parasitica* | X | X |
| *Aphanocapsa* sp. | X |  |
| *Aphanothece* sp*.* | X |  |
| *Chroococcus dispersus* | X | X |
| *Chroococcus giganteus* |  | X |
| *Chroococcus limneticus* |  | X |
| *Chroococcus minutus* | X | X |
| *Cuspidothrix* cf. *issatschenkoi* | X | X |
| *Cylindrospermopsis raciborskii* | X | X |
| *Dolichospermum* cf. *flos-aquae* |  | X |
| *Dolichospermum lemmermannii* | X | X |
| *Dolichospermum planctonica* | X | X |
| *Dolichospermum* sp. | X |  |
| *Geitlerinema amphibium* | X |  |
| *Jaaginema subtilissimum* | X | X |
| *Limnothrix* cf. *putrida* |  | X |
| *Limnothrix redekei* | X | X |
| *Merismopedia glauca* |  | X |
| *Merismopedia minima* |  | X |
| *Merismopedia tenuissima* |  | X |
| *Oscillatoria limosa* |  | X |
| *Oscillatoria sancta* |  | X |
| *Oscillatoria* sp. | X | X |
| *Oscillatoria tenuis* |  | X |
| *Phormidium* sp. | X | X |
| *Planktolyngbya limnetica* | X | X |
| *Pseudanabaena catenata* | X | X |
| *Raphidiopsis mediterranea* | X | X |
| *Snowella lacustris* | X |  |
| *Sphaerospermopsis aphanizonemoides* | X |  |
| *Trichodesmium* cf. *lacustre* |  | X |
| *Tychonema* cf. *bornetti* |  | X |
| *Woronichinia sp.* |  | X |
| **Kl. Chrysophyceae** |  |  |
| *Chrysophycean cyst* | X | X |
| *Dinobryon cylindricum* | X | X |
| *Dinobryon divergens* | X | X |
| *Dinobryon korsikovii* |  | X |
| *Dinobryon sertularia* | X | X |
| *Dinobryon sociale* | X | X |
| Small Flagellated chrysophycean n.i. | X | X |
| *Synura* sp. |  | X |
| **Kl. Xanthophyceae** |  |  |
| *Centritractus belenophorus* | X |  |
| *Goniochloris fallax* | X | X |
| *Goniochloris pseudogigas* |  | X |
| *Goniochloris* sp*.* | X |  |
| *Ophiocytium* cf*. capitatum* | X | X |
| *Tetraplektron laevis* | X | X |
| **Bacillariophyceae** |  |  |
| *Acanthoceras zachariasii* | X | X |
| *Achnanthidium minutissima* | X | X |
| *Amphipleura pellucida* |  | X |
| *Amphora* cf. *ovalis* | X | X |
| *Amphora libyca* | X | X |
| *Amphora* sp. |  | X |
| *Anomoeoneis sphaerophora* | X |  |
| *Asterionella formosa* | X |  |
| *Aulacoseira* cf. *islandica* |  | X |
| *Aulacoseira granulata* | X | X |
| *Aulacoseira* sp. | X | X |
| *Caloneis* sp. | X | X |
| *Centric diatoms (spp. <5 µm)* | X | X |
| *Cocconeis pediculus* | X |  |
| *Cocconeis placentula* | X | X |
| *Cyclotella meneghiniana* | X | X |
| *Cymatopleura inaequalis* | X | X |
| *Cymbella caespitosum* | X | X |
| *Cymbella lanceolata* | X | X |
| *Cymbella* sp. |  | X |
| *Diatoma ehrenberghii* | X |  |
| *Diatoma moniliformis* | X |  |
| *Diatoma tenuis* | X |  |
| *Diatoma vulgaris* | X |  |
| *Diploneis* sp*.* |  | X |
| *Ellerbeckia cf. arenaria* | X |  |
| *Encyonema minutum* | X | X |
| *Epithemia sorex* | X | X |
| *Eucocconeis laevis* |  | X |
| *Eunotia* sp*.* | X | X |
| *Fragilaria acus* |  | X |
| *Fragilaria brevistriata* |  | X |
| *Fragilaria capucina* | X | X |
| *Fragilaria crotonensis* |  | X |
| *Gomphonema acuminatum* | X | X |
| *Gomphonema affine* |  | X |
| *Gomphonema olivaceum* | X |  |
| *Gomphonema truncatum* | X | X |
| *Gyrosigma attenuatum* | X | X |
| *Hannaea arcus* | x | X |
| *Hippodonta capitata* | X | X |
| *Mastogloia smithii* | X |  |
| *Melosira varians* | X | X |
| *Meridion circulare* | X | X |
| *Navicula capiatoradiata* | X | X |
| *Navicula cf. protracta* | X |  |
| *Navicula cuspidata* | X | X |
| *Navicula menisculus* | X | X |
| *Navicula radiosa* | X | X |
| *Navicula* sp. |  | X |
| *Navicula trivialis* | X | X |
| *Neidium* sp*.* | X |  |
| *Nitzschia acicularis* | X | X |
| *Nitzschia amphibia* | X | X |
| *Nitzschia angustata* | X | X |
| *Nitzschia linearis* | X | X |
| *Nitzschia palea* | X | X |
| *Nitzschia reversa* | X |  |
| *Nitzschia sigma* |  | X |
| *Nitzschia sigmoidea* | X | X |
| *Pinnularia major* | X | X |
| *Pinnularia* sp. | X | X |
| *Rhoicosphenia abbreviata* | X | X |
| *Rhopalodia gibba* | X | X |
| *Sellaphora pupula* | X | X |
| *Skeletonema* potamos | X | X |
| *Staurosira construens* | X | X |
| *Staurosirella leptostauron* | X | X |
| *Staurosirella pinnata* | X | X |
| *Stephanodiscus hantzschii* | X |  |
| *Surirella cf. linearis* |  | X |
| *Surirella librile* |  | X |
| *Surirella ovalis* | X |  |
| *Terpsinoe* sp. |  | X |
| *Tryblionella levidensis* | X |  |
| *Ulnaria capitata* |  | X |
| *Ulnaria ulna* | X | X |
| **Kl. Cryptophyceae** |  |  |
| *Chilomonas* sp. | X |  |
| *Chroomonas* sp. | X | X |
| *Cryptomonas* cf. *curvata* | X | X |
| *Cryptomonas erosa* | X | X |
| *Cryptomonas marssonii* | X | X |
| *Plagioselmis* sp. | X | X |
| **Kl. Dinophyceae** |  |  |
| *Ceratium cornutum* | X |  |
| *Ceratium hirundinella* | X | X |
| *Glenodinium* sp. | X |  |
| *Gymnodinium* spp. | X | X |
| *Peridinium* spp. | X | X |
| **Kl. Euglenophyceae** |  |  |
| *Cryptoglena skujae* | X | X |
| *Euglena agilis* | X | X |
| *Euglena caudata* | X | X |
| *Euglena deses* | X | X |
| *Euglena ehrenbergii* |  | X |
| *Euglena gracilis* | X | X |
| *Euglena limnophila* | X |  |
| *Euglena navicula* | X |  |
| *Euglena* sp. | X | X |
| *Euglena texta* |  | X |
| *Lepocinclis acus* | X | X |
| *Lepocinclis fusiformis* | X | X |
| *Lepocinclis ovum* | X | X |
| *Lepocinclis oxyuris* | X | X |
| *Lepocinclis tripteris* |  | X |
| *Monomorphina pyrum* | X | X |
| *Phacus curvicauda* | X | X |
| *Phacus longicauda* | X | X |
| *Phacus orbicularis* |  | X |
| *Phacus* sp. |  | X |
| *Phacus tortus* |  | X |
| *Strombomonas fluviatilis* | X | X |
| *Strombomonas urceolata* | X |  |
| *Trachelomonas armata* |  | X |
| *Trachelomonas cf. rotunda* |  | X |
| *Trachelomonas hispida* | X | X |
| *Trachelomonas oblonga var. pulcherrima* |  | X |
| *Trachelomonas planctonica* | X | X |
| *Trachelomonas rugulosa* |  | X |
| *Trachelomonas* sp*.* |  | X |
| *Trachelomonas verrucosa* | X | X |
| *Trachelomonas volvocina* | X | X |
| **Kl. Chlorophyceae** |  |  |
| *Actinastrumhantzschii* | X | X |
| *Ankistrodesmus fusiformis* | X | X |
| *Ankistrodesmus falcatus* |  |  |
| *Ankyra judayi* | X |  |
| *Chlamydomonas sp.* |  | X |
| Chlorococcalean n.i. |  | X |
| *Chlorogonium* sp. | X |  |
| *Coelastrum microporum* | X | X |
| *Coelastrum reticulatum* | X |  |
| *Coenochloris fottii* | X | X |
| *Coenochloris planktonicus* | X |  |
| *Coeslastrum astroideum* |  | X |
| *Coronastrum cf. chodatti* | X |  |
| *Crucigenia quadrata* | X | X |
| *Crucigenia tetrapedia* | X | X |
| *Crucigeniella crucifera* | X | X |
| *Desmodesmus bicaudatus* | X | X |
| *Desmodesmus brasiliensis* | X | X |
| *Desmodesmus cf. pannonicus* |  | X |
| *Desmodesmus dispar* |  | X |
| *Desmodesmus intermedius* | X | X |
| *Desmodesmus lefevrei* |  | X |
| *Desmodesmus opoliensis* | X | X |
| *Desmodesmus sempervirens* | X | X |
| *Desmodesmus spinosus* | X | X |
| *Dictyosphaerium ehrenbergianum* | X | X |
| *Dictyosphaerium pulchellum* | X | X |
| *Dictyosphaerium tetrachotomum* | X | X |
| *Didymocystis* sp. | X | X |
| *Didymogenes palatina* | X |  |
| *Didympocystis inermis* |  | X |
| *Diplochloris lunata* | X |  |
| *Fusola* viridis | X | X |
| *Geminella* spp*.* | X | X |
| *Golenkinia brevispina* | X | X |
| *Golenkinia radiata* |  | X |
| *Golenkiniopsis solitaria* |  | X |
| *Granulocystopsis* sp. |  | X |
| *Keratococcus* sp. |  | X |
| *Kirchneriella irregularis* | X | X |
| *Lagerheimia genevensis* |  | X |
| *Microspora* sp. | X |  |
| *Monoraphidium caribeum* | X | X |
| *Monoraphidium circinale* | X | X |
| *Monoraphidium contortum* | X | X |
| *Monoraphidium griffithii* | X | X |
| *Monoraphidium komarkovae* | X | X |
| *Monoraphidium minutum* | X | X |
| *Monoraphidium tortile* | X |  |
| *Nephrocytium agardhianum* | X | X |
| *Nephroselmis sp.* | X | X |
| *Oocystis borgei* | X | X |
| *Oocystis lacustris* | X | X |
| *Pediastrum boryanum* | X | X |
| *Pediastrum duplex* | X | X |
| *Pediastrum simplex* | X | X |
| *Pediastrum tetras* | X | X |
| *Phacotus lenticularis* | X |  |
| *Planktonema* sp. | X | X |
| *Planktosphaeria gelatinosa* |  | X |
| *Raysiella* sp. |  | X |
| *Scenedesmus acuminatus* | X | X |
| *Scenedesmus ecornis* | X | X |
| *Scenedesmus linearis* | X | X |
| *Scenedesmus obtusus* | X | X |
| *Scenedesmus smithii* | X | X |
| *Schroederia* spp. |  | X |
| *Selenastrum bibraianum* |  | X |
| *Spermatozopsis exsultans* | X |  |
| *Tetradesmus* sp. |  | X |
| *Tetraedron caudatum* | X | X |
| *Tetraedron minimum* | X | X |
| *Tetraedron triangulare* | X | X |
| *Tetrastrum cf triangulare* | X | X |
| *Tetrastrum komarekii* | X | X |
| *Tetrastrum staurogeniiforme* | X | X |
| *Treubaria schmidlei* |  | X |
| *Treubaria triappendiculata* | X |  |
| *Ulothrix* sp*.* |  | X |
| *Westella botryoides* |  | X |
| **Kl. Zygnematophyceae** |  |  |
| *Closterium aciculare* | X |  |
| *Closterium dianae* |  | X |
| *Closterium ehrenbergii* | X | X |
| *Closterium limneticum* | X | X |
| *Closterium moniliferum* | X | X |
| *Closterium parvulum* | X |  |
| *Cosmarium botrytis* | X | X |
| *Cosmarium* cf. *impressulum* |  | X |
| *Cosmarium depressum* | X | X |
| *Cosmarium laeve* |  | X |
| *Cosmarium moniliforme* | X |  |
| *Cosmarium ornatum* |  | X |
| *Cosmarium praegrande* | X |  |
| *Cosmarium reniforme* |  | X |
| *Cosmarium* sp. | X | X |
| *Euastrum* cf. *evolutum* |  | X |
| *Spirogyra* sp. |  | X |
| *Spondylosium cf planum* |  | X |
| *Staurastrum dejectum* |  | X |
| *Staurastrum leptocladum* |  | X |
| *Staurastrum paradoxum* |  | X |
| *Staurastrum polimorphum* |  | X |
| *Staurastrum* sp. | X | X |
| *Zygnema* sp. | X | X |
|  |  |  |
| ***ZOOPLANKTON*** |  |  |
| ***Rotifera*** |  |  |
| *Anuaeropsis fissa* | X | X |
| *Ascomorpha saltans* | X | X |
| *Ascomorpha agilis* | X | X |
| *Asplanchna priodonta* | X | X |
| *Asplachna intermedia* | X |  |
| *Asplachna sieboldii* |  | X |
| *Bdelloidea* | X |  |
| *Brachionus angularis* | X | X |
| *Brachionus diversicornis* | X |  |
| *Brachionus bennini* |  | X |
| *Brachionus calyciflorus* | X | X |
| *Brachionus forficula* | X | X |
| *Brachionus havanaensis* | X |  |
| *Brachionus falcatus* |  | X |
| *Brachionus leydigii* | X |  |
| *Brachionus mirus* | X |  |
| *Brachionus patulus* | X | X |
| *Brachionus quadridentata* | X | X |
| *Brachionus urceolaris* |  | X |
| *Cephalodella forficula* | X |  |
| *Cephalodella gibba* | X | X |
| *Cephalodella xenica* | X | X |
| *Cephalodella* sp. | X | X |
| *Collotheca ambigua* | X | X |
| *Colurella adriatica* | X | X |
| *Colurella colurus* | X | X |
| *Colurella uncinata* | X | X |
| *Conochilus unicornis* | X | X |
| *Dicranophorus* sp. |  | X |
| *Epiphanes* sp. |  | X |
| *Euchlanis dilatata* | X | X |
| *Euchlanis triquetra* | X |  |
| *Euchlanis incisa* |  | X |
| *Filinia limnetica* | X |  |
| *Filinia braquiata* | X |  |
| *Filinia longiseta* |  | X |
| *Kellicottia longispina* | X |  |
| *Keratella cochlearis* | X | X |
| *Keratella tecta* | X | X |
| *Keratella quadrata* | X | X |
| *Lecane bifurca* | X |  |
| *Lecane flosculosa* |  | X |
| *Lecane bryophila* | X |  |
| *Lecane bulla* | X | X |
| *Lecane closterocerca* | X | X |
| *Lecane cornuta* | X |  |
| *Lecane copeis* |  | X |
| *Lecane curvicornis* | X |  |
| *Lecane elongata* | X | X |
| *Lecane flexilis* | X | X |
| *Lecane elsa* |  | X |
| *Lecane furcata* | X | X |
| *Lecane hamata* | X | X |
| *Lecane lunaris* | X | X |
| *Lecane luna* |  | X |
| *Lecane nana* | X |  |
| *Lecane ludwigii* |  | X |
| *Lecane papuana* | X |  |
| *Lecane pyriformis* | X | X |
| *Lecane quadridentata* | X |  |
| *Lecane quadricornis* |  | X |
| *Lecane sculata* |  | X |
| *Lecane stenroosi* | X | X |
| *Lecane stichaea* | X | X |
| *Lecane subtilis* | X |  |
| *Lecane ungulata* | X |  |
| *Lecane* sp. | X | X |
| *Lepadella acuminata* | X | X |
| *Lepadella imbricata* | X |  |
| *Lepadella patella* | X | X |
| *Lepadella triptera* | X | X |
| *Lepadella rhomboides* | X | X |
| *Lepadella rhomboidula* |  | X |
| *Lepadella* sp. | X | X |
| *Lindia* sp. | X |  |
| *Lophocaris* sp. | X | X |
| *Macrochaetus* sp. | X | X |
| *Monommata* sp. | X | X |
| *Notholca acuminata* |  | X |
| *Mytilina ventralis* | X | X |
| *Mytilina bisulcata* | X |  |
| *Platyias quadricornis* |  | X |
| *Plationus patulus* |  | X |
| *Ploesoma hudsoni* |  | X |
| *Polyarthra dolychoptera* | X | X |
| *Polyarthra longiremis* |  | X |
| *Polyarthra luminosa* |  | X |
| *Polyarthra major* | X | X |
| *Polyarthra remata* | X | X |
| *Polyarthra vulgaris* | X |  |
| *Pompholix* sp. | X | X |
| *Ptygyra* sp. |  | X |
| *Scaridium longicaudum* | X | X |
| *Sinantherina* sp. | X |  |
| *Squatinella mutica* | X | X |
| *Synchaeta* sp. | X | X |
| *Testudinella patina* | X | X |
| *Testudinella ohlei* | X | X |
| *Trichocerca bicristata* | X | X |
| *Trichocerca bidens* | X |  |
| *Trichocerca brachyura* | X | X |
| *Trichocerca capucina* |  | X |
| *Trichocerca elongata* | X | X |
| *Trichocerca dixonuttali* |  | X |
| *Trichocerca iernis* | X | X |
| *Trichocerca insulana* | X |  |
| *Trichocerca longiseta* | X |  |
| *Trichocerca pusilla* | X | X |
| *Trichocerca porcellus* |  | X |
| *Trichocerca rattus* | X | X |
| *Trichocerca similis* | X | X |
| *Trichocerca tenuior* | X | X |
| *Trichocerca weberi* | X | X |
| *Trichocerca pocillum* | X | X |
| *Trichocerca* sp. | X | X |
| *Trichotria tetractis* | X | X |
| ***Crustacea*** |  |  |
| ***Cladocera*** |  |  |
| *Alona quadrangula* | X | X |
| *Alona rectangula* | X |  |
| *Alona costata* | X | X |
| *Alona* sp. | X | X |
| *Alonella nana* | X |  |
| *Acroperus* sp. | X | X |
| *Bosmina longirostris* | X | X |
| *Chydorus sp.* | X | X |
| *Ceriodaphnia quadrangula* | X | X |
| *Ceriodaphnia reticulata* |  | X |
| *Daphnia cucullata* | X | X |
| *Daphnia longispina* |  | X |
| *Diaphanosoma brachyurum* | X | X |
| *Diaphanosoma orghidani* | X | X |
| *Eurycercus lamellatus* | X |  |
| *Graptoleberis sp.* |  | X |
| *Illyocriptus* sp. | X |  |
| *Leptodora* *kindti* | X |  |
| *Macrothrix rosea* | X | X |
| *Macrothrix laticornis* |  | X |
| *Moina micrura* | X | X |
| *Onchobunops* sp. | X |  |
| *Pleuroxus aduncus* | X |  |
| *Pleuroxus denticulatus* | X | X |
| *Pleuroxus truncatus* | X | X |
| *Pleuroxus uncinatus* | X |  |
| *Polyphemus pediculus* | X | X |
| *Scapholeberis mucronata* | X | X |
| *Sida crystallina* | X | X |
| *Simocephalus punctatus* |  | X |
| *Simocephalus serrulatus* | X | X |
| *Simocephalus vetulus* | X | X |
| ***Copepoda*** |  |  |
| *Acanthocyclops americanus* | X | X |
| *Acanthocyclops robustus* | X | X |
| *Eucyclops macrunoides* | X | X |
| *Eucyclops serrulatus* | X |  |
| *Macrocyclops albidus* | X | X |
| *Macrocyclops fuscus* |  | X |
| *Mesocyclops leuckarti* | X | X |
| *Cryptocyclops bicolor* | X | X |
| *Thermocyclops oithonoides* | X | X |

*List of identification keys used for phytoplankton species determination. The Algae base was used to check the species names that are currently accepted taxonomically.*

Guiry, M.D. & Guiry, G.M. (2017). AlgaeBase. World-wide electronic publication, National University of Ireland, Galway. http://www.algaebase.org; searched on 12 October 2017.

Huber-Pestalozzi, G. (1955). Das Phytoplankton des Süßwassers, Systematik und Biologie, 4.Teil: Euglenophyceen. In: A. Thienemann (Ed.), Die Binnengewässer, Band 16, 4. Teil. E. Schweizbart’sche Verlagsbuchhandlung, Stuttgart.

Komarek, J. & Fott. B. (1983). Chlorophyceae (Grünalgen), Ordnung: Chlorococcales. In: G. Huber-Pestalozzi (Ed.), Das Phytoplankton des Süßwassers, Heft 7/1. Schweitzerbart’sche Verlagsbuchhandlung, Stuttgart.

Komarek, J. & Anagnostidis K. (1998). Cyanoprokaryota. 1. Teil Chroococcales. In: H. Ettl, G. Gärtner, H. Heynig& G. Anagnostidis (Eds), Süßwasserflora von Mitteleuropa, Band 19/1. G.FischerVerlag, Stuttgart – New York.

Komarek, J. & Anagnostidis K. (2005). Cyanoprokaryota. 2. Teil Oscillatoriales. In: H. Büdel, G. Gärtner, L. Krienitz& M. Schagerl (Eds), Süßwasserflora von Mitteleuropa, Band 19/2. G.FischerVerlag, Stuttgart – New York.

Komarek, J. (2013) Cyanoprokaryota. 3. Teil Heterocytous Genera. In: Süßwasserflora von Mitteleuropa, Band 19/3. (EdsH. Büdel, G. Gärtner, L. Krienitz& M. Schagerl), G.FischerVerlag, Stuttgart – New York.

Krammer K. & Lange-Bertalot H. (1986) Bacillariophyceae. 1: Teil: Naviculaceae. In: Sußwasserflora von Mitteleuropa, Band2 ⁄ 1 (Eds H. Ettl, G. Gartner, J. Gerloff, H. Heynig& D.Mollenhauer), 876 pp. Gustav Fischer Verlag, Stuttgart ⁄ New York.

Krammer K. & Lange-Bertalot H. (1988) Bacillariophyceae.2:Teil: Bacillariaceae, Epithmiaceae, Surirellaceae. In: Sußwasserflora von Mitteleuropa, Band 2 ⁄ 2 (Eds H. Ettl, G.Gartner, J. Gerloff, H. Heynig & D. Mollenhauer), 610 pp.Gustav Fischer Verlag, Stuttgart ⁄ New York.

Krammer K. & Lange-Bertalot H. (1991a) Bacillariophyceae.3: Teil: Centrales, Fragilariaceae, Eunotiaceae. In: Sußwasserflora von Mitteleuropa, Band 2 ⁄ 3 (Eds H. Ettl, G. Gartner, J.Gerloff, H. Heynig& D. Mollenhauer), 576 pp. GustavFischer Verlag, Stuttgart ⁄ Jena

Krammer K. & Lange-Bertalot H. (1991b) Bacillariophyceae. 4: Teil: Achnanthaceae. In: Sußwasserflora von Mitteleuropa,Band 2 ⁄ 4 (Eds H. Ettl, G. Gartner, J. Gerloff, H. Heynig& D.Mollenhauer), 437 pp. Gustav Fischer Verlag, Stuttgart ⁄ Jena.

Kristiansen J. & Gärtner H. (2007) Chrysophyte and Haptophyte Algae: Synurophyceae, Band 01:02: (Eds. Burkhard Bürgel, Georg Gärtner, Lothar Krienitz, Hans R. Preisig und Michael Schagerl).

Novarino, G. (2003). A companion to the identification of cryptomonad flagellates (Cryptophyceae = Cryptomonadea). *Hydrobiologia*, 502, 225–270.

Figures legend

Figure S1. Species accumulation curves for a. phytoplankton; b. rotifers and c. microcrustaceans in post-flood and low water conditions.

Figure S2. Results of the variation partitioning (db-RDA) of community composition (abundance data) into environmental (Env), spatial factors at the section (Sec) and wetland scales (Wet) in post-flood (a-c) and low water (d) conditions. The contribution of each factor is represented by R^2^ Adjusted values, bold numbers indicate significant effects (p<0.05). Negative values are not shown.
